# Modification and Validation of an mHealth App Quality Assessment Methodology for International Use: Cross-sectional and eDelphi Studies

**DOI:** 10.2196/36912

**Published:** 2022-08-19

**Authors:** Fionn Woulfe, Kayode Philip Fadahunsi, Michael O'Grady, Griphin Baxter Chirambo, Mala Mawkin, Azeem Majeed, Simon Smith, Patrick Henn, John O'Donoghue

**Affiliations:** 1 School of Medicine University College Cork Cork Ireland; 2 Department of Primary Care and Public Health Imperial College London London United Kingdom; 3 School of Computer Science University College Dublin Dublin Ireland; 4 Faculty of Health Sciences Mzuzu University Mzuzu Malawi; 5 School of Medicine Imperial College London London United Kingdom; 6 ASSERT Research Centre School of Medicine University College Cork Cork Ireland; 7 Malawi eHealth Research Centre University College Cork Cork Ireland

**Keywords:** evaluation tool, mobile health, mHealth, smartphone app, app, international mHealth

## Abstract

**Background:**

Over 325,000 mobile health (mHealth) apps are available to download across various app stores. However, quality assurance in this field of medicine remains relatively undefined. Globally, around 84% of the population have access to mobile broadband networks. Given the potential for mHealth app use in health promotion and disease prevention, their role in patient care worldwide is ever apparent. Quality assurance regulations both nationally and internationally will take time to develop. Frameworks such as the Mobile App Rating Scale and Enlight Suite have demonstrated potential for use in the interim. However, these frameworks require adaptation to be suitable for international use.

**Objective:**

This study aims to modify the Enlight Suite, a comprehensive app quality assessment methodology, to improve its applicability internationally and to assess the preliminary validity and reliability of this modified tool in practice.

**Methods:**

A two-round Delphi study involving 7 international mHealth experts with varied backgrounds in health, technology, and clinical psychology was conducted to modify the Enlight Suite for international use and to improve its content validity. The Modified Enlight Suite (MES) was then used by 800 health care professionals and health care students in Ireland to assess a COVID-19 tracker app in an online survey. The reliability of the MES was assessed using Cronbach alpha, while the construct validity was evaluated using confirmatory factor analysis.

**Results:**

The final version of the MES has 7 sections with 32 evaluating items. Of these items, 5 were novel and based on consensus for inclusion by Delphi panel members. The MES has satisfactory reliability with a Cronbach alpha score of .925. The subscales also demonstrated acceptable internal consistency. Similarly, the confirmatory factor analysis demonstrated a positive and significant factor loading for all 32 items in the MES with a modestly acceptable model fit, thus indicating the construct validity of the MES.

**Conclusions:**

The Enlight Suite was modified to improve its international relevance to app quality assessment by introducing new items relating to cultural appropriateness, accessibility, and readability of mHealth app content. This study indicates both the reliability and validity of the MES for assessing the quality of mHealth apps in a high-income country, with further studies being planned to extrapolate these findings to low- and middle-income countries.

## Introduction

Use and access to mobile phones and the internet is ubiquitous in many countries [1]. In 2020, there were 4 billion mobile internet users, and this figure is expected to grow to 5 billion by 2025 [2]. In 2017, over 325,000 mobile health (mHealth) apps were available to download across various app stores with the number of app publishers rising by 45% in the same year [3]. This market proliferation has created a challenging task for health care professionals to identify high-quality apps, as many have been created without expert medical involvement, appropriate testing, and validation [4]. A review published in 2020 indicated that most safety concerns about apps related to the quality of their content [5]. Examples of inappropriate app content include a recommendation for people with bipolar disorder to “take a shot of hard liquor an hour before bed” and a suggestion that bipolar disorder is “contagious” [6].

Given the rapid proliferation of mHealth apps, regulation of this sector is challenging for policy makers [7]. Various strategies are being used to tackle shortcomings of mHealth apps especially in high-income countries (HICs). For example, the Food and Drug Administration applies regulatory oversight to a subgroup of mHealth apps regarded as medical devices or that pose patient safety risks [8]. For low- and middle-income countries (LMIC), there is a growing demand to develop and apply assessment frameworks that meet contextual aspects relevant to one’s specific country. While comprehensive, timely, and effective national regulation is awaited, various mHealth app quality assessment methodologies have been proposed for use in the interim. Examples include the Enlight Suite [9], the Mobile App Rating Scale [10], and the App Chronic Disease Checklist (ACDC) [11].

A review of mHealth app quality assessment methodologies indicated the scope for improvement of such methodologies to enhance their comprehensiveness and relevance across resource-diverse settings [12]. The review found that none of the existing generic app assessment methodologies [9-11,13,14] explicitly considered cultural appropriateness. Only two methodologies addressed privacy and security of information [9,13]. Similarly, readability was considered by only two methodologies [11,13]. Only the ACDC [11] addressed the availability of mHealth apps in offline mode. The ability of the apps to facilitate behavior change was only addressed by three methodologies [9-11]. In addition, most existing generic app assessment methodologies only offered some form of face and content validity based on expert opinions [9-11,13] with the reliability of only 2 methodologies reported [9,10]. The construct validity of all the app assessment methodologies was not evaluated [12].

Although the Enlight Suite was adjudged as thorough and comprehensive, it has limited international applicability because it does not consider attributes that are relevant to the successful uptake of mHealth apps in low-, middle-, and HICs, including cultural appropriateness, readability, and access [12,15]. It is important to consider cultural appropriateness when developing content and designing user interfaces of apps for international and country-specific audiences [16]. If the content or user interface of an mHealth app is not culturally appropriate for a particular audience, acceptability and uptake may be low [16]. Similarly, poor readability may affect the acceptability and uptake of apps among prospective users [17,18]. Previous research revealed that many mHealth apps were written at excessively high reading grade levels, which may not be suitable for users with low levels of literacy especially in LMIC [17-19]. In addition, access to the internet may affect mHealth use especially in LMIC and among deprived communities of HICs [20]. Although the mobile broadband penetration rate has doubled in LMIC over the last decades [21], users continue to experience challenges with the cost and speed of internet services.

The purpose of this study is therefore to modify the Enlight Suite [9] to be more considerate and effective for use internationally. Additionally, this paper serves to provide an initial reliability and validity assessment of the Modified Enlight Suite (MES) in practice.

## Methods

### Verifying the Content Validity of the Modified Enlight Suite

To formulate the MES and confirm content validity, a two-round iterative Delphi process was undertaken. Delphi techniques are widely used for this type of research with its validity for questionnaire formulation and modification confirmed in past literature [22,23].

### Participant Characteristics and Recruitment

Previous research recommends having between 3 and 10 professionals to verify content validity [24]. Therefore, a total of 7 digital health researchers with backgrounds in clinical medicine (n=4), nursing (n=1), clinical psychology (n=1), and information technology (n=1) were recruited in this phase of the study. Of the participants, 3 were affiliated universities in Ireland, 1 was affiliated to a university in Malawi, and 3 were affiliated to universities in the United Kingdom. Although most of these experts currently reside in HICs, they have varied hands-on clinical (n=2) or research (n=5) experience in LMIC.

Experts were identified based on the following inclusion criteria: hold a professional title in the areas of technology, medicine, health, or clinical psychology; have a minimum of 2 years professional experience in their respective field; be willing to engage in all Delphi phases of this study digitally; and have suitable internet access.

### Delphi Process

The panel of experts analyzed the questions in the pre-existing Enlight Suite as well as those proposed by the facilitators (FW and JOD). Version 1 (V1) of the MES contained 7 sections with a total of 33 questions. Of these questions, the facilitators proposed 5 questions based on considerations of a past review of app assessment methodologies indicating potential weaknesses in the Enlight Suite for international mHealth app evaluation practices [12]. Each panelist was asked to examine the suitability of questions within V1 of the MES for mHealth app evaluation practices internationally, both individually and collectively. Participants were asked to consider each of the questions (and proposed questions) with respect to its appropriateness and relevance across all resource-level settings (ie, HICs and LMIC).

During round 1 of the Delphi process, both quantitative and qualitative feedback were gathered. For quantitative evaluation of the content validity, a 3-point scale was used (1=“exclude question,” 2=“include question but modify,” and 3=“include question as is”) to rate each question. Whenever a panelist indicated that a question should be modified, qualitative feedback was requested. Additionally, panelists were asked at the end of each section of the MES if further adaptations to that section were necessary.

Standard methods to determine consensus in Delphi studies are not available [25]. However, for the purposes of this study, consensus was measured via the percentage who agreed with amendments after round 1 (≥4/7). Following round 1 of the Delphi process, the facilitators (FW and JOD) discussed suggested amendments and reflected on both qualitative and quantitative feedback before formulating Version 2 (V2) of the MES. Results were summarized and panelist feedback was anonymized before round 2 of the Delphi process commenced.

In round 2, panelists were provided with V2 of the MES. During this round, panelists were asked if they accepted or rejected the changes that the facilitators made to V1 of the MES to create V2. Additionally, panelists could review comments and suggestions made by fellow participants, albeit anonymously. The Delphi process would be terminated should the outcome of a round yield “minor” or “out of scope” amendments only. In this case, the facilitators would discuss the feedback and make changes accordingly without another round occurring.

### Verification of the Validity and Reliability of the Modified Enlight Suite

To assess the reliability of the MES, the construct was distributed in digitized form to participants who were asked to use it to evaluate the Irish COVID-19 app, a popular freely available mHealth app in Ireland [26]. The MES was tested in Ireland to serve two purposes: (1) to avoid language acting as a confounding variable falsely affecting reliability results and (2) for convenience purposes to promptly identify reliability issues prior to international testing.

The following were inclusion criteria to participate: be a health care professional or health care student with a minimum of 2 years clinical exposure, own a smartphone device, and be familiar with the Irish COVID-19 app.

Convenience sampling was used to recruit participants via targeted social media platforms and through the university emailing list. When validating a questionnaire, there are no fixed rules for an ideal sample size [27]. Some have suggested that a sample size of 50 is considered very poor, 200 as fair, and >1000 being excellent [28]. Larger sample sizes are always more reflective of the population; ergo, the investigators sought as many participants as possible.

**Figure 1:**
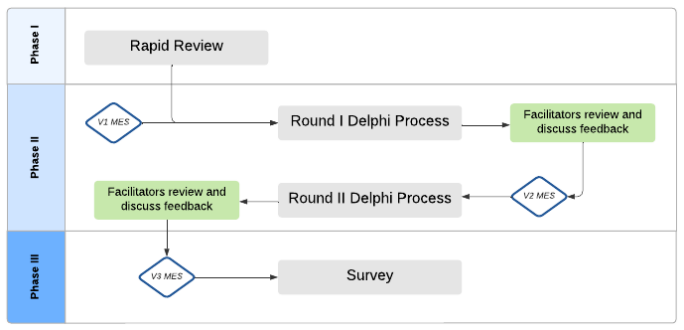
Flow Chart Indicating Each Phase of the Research Process. MES: Modified Enlight Suite.

The reliability of the MES was assessed using SPSS version 28 software (IBM Corp). The Cronbach α for the overall Enlight scale and each of the seven subscales (usability, visual design, user engagement, content, therapeutic persuasiveness, therapeutic alliance, and general subjective evaluation of the app’s purpose) was calculated. A Cronbach α of .7 or above is traditionally regarded as an indication of reliability [29]. The construct validity was assessed using Amos version 26 (IBM Corp) for confirmatory factor analysis. The model for the confirmatory factor analysis was based on the seven pre-existing categories listed above. A flowchart indicating each stage of this research can be viewed in Figure 1.

### Ethical Considerations

The Social Research Ethics Committee of University College Cork Ireland granted ethical approval for this stage of the project (SREC/SOM/19062020/1/25112020/). Prior to engagement, participants were given an information leaflet with details of the study and asked to sign a consent form. All data collected during the study was kept secure on a password-encrypted computer. This research was partly funded by an Irish Health Research Board Scholarship (scholarship SS-2020-089).

## Results

### Developing the Finalized Version of the Modified Enlight Suite

#### Round 1

Round 1 of the Delphi study was conducted in July 2020. During this round, the facilitators proposed five questions to be included in the MES. Of these questions, three served to improve the relevance of the MES for quality assessment purposes internationally. These questions were based on the following topics: (1) culture appropriateness, (2) accessibility, and (3) readability. The facilitators also proposed 2 questions that affect a user’s ongoing use of an app. These questions were concerned with the following topics: (1) errors and (2) timeliness. Following round 1 of the Delphi process, consensus was reached that each of these questions should be included within the final version of the MES.

Furthermore, the panelists made 47 suggestions/comments. These were categorized by the facilitators into “minor amendments” (n=33), “significant amendments” (n=9), and “other comments” (n=5). Following this round and discussion by the facilitators, 26 of these amendments were accepted and incorporated to create V2 of the MES. An extraction table with categorized feedback from round 1 can be viewed in Multimedia Appendix 1.

#### Round 2

V2 of the MES contained 7 sections with 32 questions. All amendments made by the facilitators based on feedback from round 1 were accepted by participants in round 2. During this round, participants provided 25 additional comments/amendments that were subcategorized into “minor” (n=18) and “other” (n=7). Of these, 12 were incorporated into the final version (ie, Version 3 [V3]) of the MES (Multimedia Appendix 2). An extraction table with feedback from round 2 can be viewed in Multimedia Appendix 3.

This multi-round iterative process was terminated after round 2, as modifications in V2 of the MES were accepted by panelists. Given the nature of feedback suggested by panelists during round 2, the facilitators made additional minor amendments as necessary. The comprehensibility of the original Enlight Suite facilitated this short Delphi process. Given the interdisciplinary nature of the professional panel, the Delphi process served to confirm the content validity of the MES for international use.

### Reliability of the MES

A total of 800 responses were gathered during this phase to assess the reliability and construct validity of the MES. Of the 800 participants, 91% (n=728) fell within the 18 to 34 years of age category. Health care professionals represented 20% (n=160) of the participants, while the remaining 80% (n=640) were health care students with a minimum of 2 years clinical work experience. The majority (n=712, 89%) of participants identified as being of White/Caucasian ethnicity. Less than half (n=376, 47%) of the responses were complete.

The reliability analysis showed satisfactory internal consistency of the overall scale (Cronbach α=.93). Similarly, the subscales demonstrated high reliability except for the user engagement scale (Cronbach α=.65), which is slightly lower than the traditionally regarded reliability level (Cronbach α=.7) [29]. Deletion of items under user engagement did not improve the reliability of the subscale. The Cronbach α for the scale and the subscales are presented in Table 1.

**Table 1:** Reliability statistics.

Section	Items, n	Cronbach α
Usability	7	.82
Visual design	3	.77
User engagement	5	.65
Content	5	.80
Therapeutic persuasiveness	6	.78
Therapeutic alliance	3	.73
General subjective evaluation of the app’s purpose	3	.76
Overall scale	32	.93

### Construct Validity of the MES

The concept of fitness in confirmatory factor analysis refers to the extent to which the empirical data (eg, our survey findings) supports the construct validity of the theoretical model being tested (which is the MES in our study) [30]. The chi-square goodness-of-fit test (*χ*^2^_443_=1045.9; *P*<.001; *χ*^2^ / *df* = 2.36) indicates that our model fits modestly with the data [28]. Although a significant *P* value as in our study indicates a poor fit, this is not unexpected due to our large sample size [30]. For studies with large sample sizes, it is recommended to consider the model as highly fit with data when the *χ*^2^ / *df* is less than 2 [30]. Although the value in our model is not less than 2, this is still an acceptable value. Similarly, the comparative fit index (0.89) and Tucker-Lewis index (0.87) show that our model modestly fits with the data, as a value of at least 0.9 is required for the model fit to be deemed acceptable [31]. However, the root-mean-square error of approximation (0.041, 95% CI 0.038-0.043) indicates that our model is a close-fitting model, as it is below the 0.05 cutoff point [32] and all factor loadings are positive and statistically significant (Table 2). In other words, the data from the survey provides support, albeit modestly, for the validity of the constructs (ie, 32 items and 7 categories) of the MES. It is worth noting that the first item in each category does not include significance tests (SE, critical ratio, and *P* value) because the unstandardized estimate for each first item was fixed at 1 rather than estimated as part of the adopted methodology, hence the empty cells in Table 2.

**Table 2:** Construct validity.

Variable		Standardized estimate	Unstandardized estimates	SE	Critical ratio	*P* value
**Usability**						
	Navigation	0.779	1	N/A^a^	N/A	N/A
	Access	0.446	0.850	0.090	9.442	<.001
	Understandability	0.653	0.827	0.058	14.256	<.001
	Errors	0.557	0.941	0.079	11.956	<.001
	Timeliness	0.670	0.976	0.067	14.663	<.001
	Learnability	0.689	0.864	0.057	15.135	<.001
	Ease of use	0.728	1.014	0.063	16.048	<.001
**Design**						
	Aesthetics	0.701	1	N/A	N/A	N/A
	Layout	0.789	1.133	0.079	14.263	<.001
	Size	0.689	0.914	0.071	12.888	<.001
**Engagement**						
	Content presentation	0.655	1	N/A	N/A	N/A
	Interactive	0.567	1.271	0.121	10.513	<.001
	Not irritating	0.459	0.941	0.108	8.705	<.001
	Targeted tailored personalized	0.455	0.966	0.112	8.629	<.001
	Captivating	0.574	1.037	0.098	10.627	<.001
**Content**						
	Evidence-based content	0.642	1	N/A	N/A	N/A
	Cultural appropriateness	0.673	1.114	0.096	11.599	<.001
	Quality of information provision	0.715	1.095	0.090	12.128	<.001
	Clarity about the app purpose	0.608	0.939	0.088	10.693	<.001
	Complete and concise	0.701	1.076	0.090	11.948	<.001
**Therapeutic persuasiveness**						
	Call to action	0.674	1	N/A	N/A	N/A
	Rewards	0.600	1.055	0.098	10.754	<.001
	Real data-driven adaptive content	0.639	0.958	0.085	11.337	<.001
	Therapeutic rationale and pathway	0.645	0.903	0.079	11.453	<.001
	Ongoing feedback	0.629	0.969	0.087	11.168	<.001
	Expectations and relevance	0.480	0.773	0.088	8.788	<.001
**Therapeutic alliance**						
	Acceptance and support	0.710	1	N/A	N/A	N/A
	Positive therapeutic expectations	0.669	0.843	0.074	11.413	<.001
	Relatability	0.676	0.972	0.084	11.502	<.001
**General subjective evaluation**						
	Appropriate features to meet the clinical aim	0.702	1	N/A	N/A	N/A
	Right mix of ability and motivation	0.674	1.002	0.083	12.086	<.001
	I like the app	0.766	1.138	0.084	13.500	<.001

^a^N/A: not applicable.

## Discussion

### Principal Findings

The objectives of this study were to modify the Enlight Suite and test the reliability and validity of the MES. The Delphi process resulted in a comprehensive MES, which contains 32 questions over 7 sections including additional dimensions not in the original Enlight Suite, including access, cultural appropriateness, readability, errors, and timeliness.

The subsequent survey demonstrated an overall reliability of the MES and its subscales. The confirmatory factor analysis demonstrated a positive and significant factor loading for all 32 items in the MES with a modestly acceptable model fit that is indicative of the construct validity of the MES.

### Comparison With Prior Work

The inclusion of cultural appropriateness, readability, and access criteria differentiates the MES from existing methodologies [9-11,13,14], which either considered only one or none of these important criteria. Questions on cultural appropriateness, readability, and access acknowledge the multi-demographic nature of the mHealth market [1]. For instance, there may be a need to present the content of an mHealth app in a local language to enhance its utility in a particular locality [1,33]. Similarly, the consideration of access in offline mode in the MES recognizes that internet access may not be continuous for many users [34]. These newly introduced dimensions (access, cultural appropriateness, and readability) have been identified by previous studies as important aspects of apps that ought to be considered for successful uptake across both HICs and LMIC [12,15]. Thus, the introduction of these dimensions has improved the applicability of the MES internationally.

The overall reliability of the MES was quite high in our study as well as the reliability of the subscales except for the user engagement subscale. However, the original Enlight Suite demonstrated adequate reliability across all domains including user engagement [9]. Interestingly, the major modifications to the Enlight Suite in this study were not in the user engagement category. The difference in the reliability results could be attributed to the variation in the approach used by the two studies. While the reliability of the original Enlight Suite was based on ratings by 2 trained researchers [9], the reliability testing in our study was based on the ratings by 800 health care professionals and students who would be the end users of the MES. Thus, the original Enlight Suite [9] was validated to be used with prior training, while the MES is validated to be used by any health care professional.

The demonstration of construct validity in this study with a modestly acceptable model fit supports the position of the authors of the original tool who regarded it as a suite consisting of multiple scales rather than a single scale whose result could be aggregated [9]. These results should be interpreted with caution due to the possible impact of the missing data in our study on the model fit. Due to missing data, we were only able to use the maximum likelihood estimation approach, which assumes that the variables are normally distributed [35].

### Strength and Limitations

This paper builds upon a rapid review that identified shortcomings of mHealth app quality assessment methodologies [12]. The MES was developed with input by international experts in mHealth. Given their diverse background and expertise, the content of this tool could be considered applicable internationally. To the best of our knowledge, the MES is the first mHealth app quality assessment methodology that considers factors known to affect the fundamental usability of mHealth technologies in LMIC.

The reliability and validity assessment of the MES in this study was undertaken in Ireland, an HIC. Of the participants who engaged in the survey, 89% (712/800) identified as either White or Caucasian. This highlights a need for similar studies to test the reliability and validity of the MES in LMIC. For the MES to be reliably effective for all, participants from more diverse backgrounds and ethnicities are needed in the future to extrapolate these findings. The modest construct validity of the MES is also a limitation, and improved modeling could possibly be achieved with less missing data.

### Future Work

While this study demonstrates the content validity via an international panel of mHealth stakeholders, health care professionals with no technological background may have been underrepresented in the Delphi process. This is currently being investigated with focus groups in Malawi and South Africa. Additional modifications may be made to V3 of the MES based on feedback from these focus groups. The reliability of the updated Enlight Suite will then be assessed with participants recruited internationally.

The original Enlight Suite provided a comprehensive quality and therapeutic potential tool for both mobile and web-based eHealth interventions. While the focus of this study was to adapt the suite to improve its international relevance for mHealth app evaluation, future works could expand on its web-based potential. This study introduced additional dimensions (access, cultural appropriateness, and readability) that are relevant to international applicability of the Enlight Suite. Future works could look into developing a framework for the international applicability of scales.

### Conclusion

The need for quality assessment in mHealth is clear. This study is a key primary step in improving the scope, content, and relevance of mHealth quality assessment methodologies across diverse settings. It is of the authors opinion that the MES is the first quality assessment methodology to also consider factors known to hinder the uptake and continued use of mHealth apps in resource-poor settings. Furthermore, the authors believe that this research improves the validity of the construct while taking measures to enhance its fundamental usability. There is scope that the MES may be adopted by health care professionals internationally to assess the quality and suitability of mHealth apps available to their patients before recommending them. This would help ensure patient safety.

## Appendix

Multimedia Appendix 1 Data extraction from round 1 of the Delphi process.

Multimedia Appendix 2 Version 3 of the Modified Enlight Suite.

Multimedia Appendix 3 Data extraction from round 2 of the Delphi process.

## Abbreviations

ACDC: App Chronic Disease Checklist
HIC: high-income country
LMIC: low- and middle-income countries
MES: Modified Enlight Suite
mHealth: mobile health
NIHR: National Institute for Health and Care Research
V1: Version 1
V2: Version 2
V3: Version 3

## References

[1] (2016). Global diffusion of eHealth: making universal health coverage achievable. Report of the third global survey on eHealth. World Health Organization.

[2] The mobile economy 2022. GSMA.

[3] mHealth app developer economics 2017: current status and future trends in mobile health. Research 2 Guidance.

[4] Cook VE, Ellis AK, Hildebrand KJ (2016). Mobile health applications in clinical practice: pearls, pitfalls, and key considerations. Ann Allergy Asthma Immunol.

[5] Akbar S, Coiera E, Magrabi F (2020). Safety concerns with consumer-facing mobile health applications and their consequences: a scoping review. J Am Med Inform Assoc.

[6] Nicholas J, Larsen ME, Proudfoot J, Christensen H (2015). Mobile apps for bipolar disorder: a systematic review of features and content quality. J Med Internet Res.

[7] Patient privacy in a mobile world: a framework to address privacy law issues in mobile health. Thomas Reuters Foundation.

[8] Larson RS (2018). A path to better-quality mHealth apps. JMIR Mhealth Uhealth.

[9] Baumel A, Faber K, Mathur N, Kane JM, Muench F (2017). Enlight: a comprehensive quality and therapeutic potential evaluation tool for mobile and web-based eHealth interventions. J Med Internet Res.

[10] Stoyanov SR, Hides L, Kavanagh DJ, Zelenko O, Tjondronegoro D, Mani M (2015). Mobile app rating scale: a new tool for assessing the quality of health mobile apps. JMIR Mhealth Uhealth.

[11] Anderson K, Burford O, Emmerton L (2016). App chronic disease checklist: protocol to evaluate mobile apps for chronic disease self-management. JMIR Res Protoc.

[12] Woulfe F, Fadahunsi K, Smith S, Chirambo G, Larsson E, Henn P, Mawkin M, O' Donoghue J (2021). Identification and evaluation of methodologies to assess the quality of mobile health apps in high-, low-, and middle-income countries: rapid review. JMIR Mhealth Uhealth.

[13] Yasini M, Beranger J, Desmarais P, Perez L, Marchand G (2016). mHealth quality: a process to seal the qualified mobile health apps. Stud Health Technol Inform.

[14] Martínez-Pérez B, de la Torre-Díez I, Candelas-Plasencia S, López-Coronado M (2013). Development and evaluation of tools for measuring the quality of experience (QoE) in mHealth applications. J Med Syst.

[15] Wallis L, Blessing P, Dalwai M, Shin SD (2017). Integrating mHealth at point of care in low- and middle-income settings: the system perspective. Glob Health Action.

[16] What is cultural appropriateness. IGI Global.

[17] Ayyaswami V, Padmanabhan DL, Crihalmeanu T, Thelmo F, Prabhu AV, Magnani JW (2019). Mobile health applications for atrial fibrillation: a readability and quality assessment. Int J Cardiol.

[18] Dunn Lopez K, Chae S, Michele G, Fraczkowski D, Habibi P, Chattopadhyay D, Donevant SB (2021). Improved readability and functions needed for mHealth apps targeting patients with heart failure: An app store review. Res Nurs Health.

[19] (2021). International student assessment (PISA). OECD iLibrary.

[20] (2018). Why do most mHealth apps fail?. Top Digital Agency.

[21] ICT Facts and Figures 2016: mobile network coverage and evolving technologies. ITU.

[22] Keeney S, Hasson F, McKenna H (2006). Consulting the oracle: ten lessons from using the Delphi technique in nursing research. J Adv Nurs.

[23] Mengual-Andrés S, Roig-Vila R, Mira JB (2016). Delphi study for the design and validation of a questionnaire about digital competences in higher education. Int J Educ Technol Higher Education.

[24] Lynn MR (1986). Determination and quantification of content validity. Nurs Res.

[25] Hsu CC, Sandford BA (2007). The Delphi technique: making sense of consensus. Practical Assess Res Eval.

[26] Health Service Executive COVID Tracker Ireland. App Store.

[27] Osborne JW, Costello AB (2004). Sample size and subject to item ratio in principal components analysis. Practical Assess Res Eval.

[28] Comfrey A, Lee H (1992). A First Course in Factor Analysis.

[29] Bolarinwa O (2015). Principles and methods of validity and reliability testing of questionnaires used in social and health science researches. Niger Postgrad Med J.

[30] Alavi M, Visentin DC, Thapa DK, Hunt GE, Watson R, Cleary M (2020). Chi-square for model fit in confirmatory factor analysis. J Adv Nurs.

[31] Byrne BM (2010). Structural Equation Modeling with AMOS: Basic Concepts, Applications, and Programming. 2nd Ed.

[32] Kline RB (2016). Principles and Practice of Structural Equation Modeling. 4th ed.

[33] Liu F, Zhao S, Li Y (2017). How many, how often, and how new? A multivariate profiling of mobile app users. J Retailing Consumer Services.

[34] Lopez-Sintas J, Lamberti G, Sukphan J (2020). The social structuring of the digital gap in a developing country. The impact of computer and internet access opportunities on internet use in Thailand. Technol Soc.

[35] Whittaker TA (2016). Structural equation modeling. Applied Multivariate Statistics for the Social Sciences. 6th ed.

