# Supplementary material for: Modification and Validation of an mHealth App Quality Assessment Methodology for International Use: Cross-sectional and eDelphi Studies
Source: JMIR Form Res. 2022 Aug 19;6(8):e36912. doi: 10.2196/36912 (PMC9497647; doi:10.2196/36912)
Supplement: Multimedia Appendix 1 [file formative_v6i8e36912_app1.docx]

| **Delphi process: Round I** |  |  |  |  |  |  |
| --- | --- | --- | --- | --- | --- | --- |
|  | **Amendments** | **Classification** |  |  | **Decision** |  |
|  |  | Minor | Major | Other | Accept | Reject |
| **Section 1: Usability** |  |  |  |  |  |  |
| Q. Navigation | Very poor. It is very difficult to move from one place to another. Many features are, therefore, not easily accessible when needed. | ✓ |  |  | ✓ |  |
| Q. Learnability | Very poor. It takes too much time to learn how to use the program. Supplementary support is needed. Poor. It takes a considerable amount of time to learn how to use the program. Only highly motivated users will take the time to learn it and supplementary support may be needed. | ✓ |  |  | ✓ |  |
| Q. Learnability | Suggest add 'intuitive' to 'Is it self-explanatory?' Would consider a description of what is meant by 'complexity' | ✓ |  |  | ✓ |  |
| Q. Ease of use | This question is too broad. It is necessary to be specific what is meant by "effort". I would think this is referring to navigation and learnability. |  |  | ✓ |  | ✓ |
| Q. Ease of use | Very poor. The user has to exert a lot of effort that would have been unnecessary had the program been better designed. 4. Good. Utilization could have been made more effortless by better designing one (not major) feature. | ✓ |  |  | ✓ |  |
| Q. Ease of use | Does the operator need to exert only the least possible effort to activate the desired features? Suggest replace with 'Does the operator need to use minimal effort to activate the desired features?' I suggest replacing 'exert' with 'use'. I suggest constancy of language 'operator' v 'user' | ✓ |  |  | ✓ |  |
| Q. Performance | I would rather use timeliness not performance because performance is too broad and non-specific. | ✓ |  |  | ✓ |  |
| Q. Errors | Errors within the app is important but it does not fit under usability category | ✓ |  |  | ✓ |  |
| Q. Readability | Comprehensibility sounds more meaningful than readability. Readability will refer font, font size, spacing etc. | ✓ |  |  | ✓ |  |
| *Other comments received at the end of this section* | Errors within the app is important but it does not fit under usability category. I would rather use timeliness not performance because performance is too broad and non-specific. Similarly, comprehensibility sounds more meaningful than readability. Readability will refer font, font size, spacing etc.  May be helpful in developing the technology to seek a hierarchy of attributes and features from end users as priorities may vary between user groups.  As regards readability or literacy levels all questions should take this into account to ensure that people with the minimal literacy skills can manage. if I recall this is to 4th class primary level. |  |  |  |  |  |
|  | If you can add a question on visual appeal. |  |  |  |  |  |
| Section 2: Visual Design |  |  |  |  |  |  |
| Q. Aesthetics | Fair. The Interface design makes some sense, but it is not attractive. 4. Attractive. Most parts of the Interface are attractive, but could be improved. 5. Very attractive. Interface design is well thought-out, and the program has a harmonious look and feel. | ✓ |  |  | ✓ |  |
| Q. Layout | Fair. For the most part, relevant elements appear on the screen and more important aspects are featured. However, there are still some issues with the structure and organization of the interface. 4. Good. In addition to ensuring the relevance and salience of key aspects, the Interface is also structured and organized. 5.Very good. The Interface is very well organized and prioritized. Elements are displayed appropriately. | ✓ |  |  |  | ✓ |
| Q. Layout | Important information' is a challenge as this tends to very between professionals and patients as reflected in the Montgomery judgement 2015. As an example the efficacy of a medication may be of less salience than the adverse effect of drowsiness to a person supporting their family as a delivery driver |  |  | ✓ |  | ✓ |
| Q. Size | Good. Most of the fonts/buttons/menus are appropriately sized, but there is at least one place where the size is wrong. | ✓ |  |  | ✓ |  |
| Q. Size | Does this address visual acuity and/ or dexterity |  |  |  |  |  |
| *Other comments received at the end of this section* | The terminology [in this section] seems inconsistent. More thought must be given to the selection of terminology. Will the average user understand the term GUI? |  | ✓ |  | ✓ |  |
|  | Aesthetic ratings may be subject to systematic bias age gender etc neutral, intuitive etc., may appeal to a wider range of users. |  |  |  |  |  |
|  | I would suggest consideration for assessing how visual impairment impacts on the use of the app. |  |  |  |  |  |
| **Section 3: User Engagement** |  |  |  |  |  |  |
| Q. Content presentation | Very good; the content presented in an engaging/interesting way. 2 Very poor. The content is poorly presented throughout. For example, there is often text where narration would be more appropriate. 4. Good. Content is delivered through an appropriate combination of features, but could be improvement. | ✓ |  |  | ✓ |  |
| Q. Interactive | Good. The program presents a good interactive experience but there is room for improvement. | ✓ |  |  | ✓ |  |
| Q. Not irritating | I suggest replace 'irritation' with 'frustration' or 'annoyance'. | ✓ |  |  |  | ✓ |
| Q. Not irritating | May stop at option 4 | ✓ |  |  |  | ✓ |
| Q. Targeted / tailored | It is a bit complex - perhaps simplified wording to be a bit more focussed? | ✓ |  |  | ✓ |  |
| Q. Captivating | Merge 13 & 17. |  | ✓ |  |  | ✓ |
| Q. Captivating | May be subject to bias i.e., age / gender thereby captivating may be regarded as aversive. |  |  | ✓ |  | ✓ |
| Q. Captivating | Replace 'pique' with 'engage' | ✓ |  |  | ✓ |  |
| Other comments received at the end of this section | You can check on the wording on irritating. If there can be another word that could mean the same but not as strong as irritating. | ✓ |  |  |  | ✓ |
|  | Q15 and Q17 could be replaced with a new question, perhaps, would you use this program? would recommend this program? or Do you find the program intuitive? Does the program do what you expect it to do? |  | ✓ |  |  | ✓ |
| **Section 4: Content** |  |  |  |  |  |  |
| Q. Evidenced based content | What does evidence-based mean? is this subjective? Again choice of terminology should be reconsidered | ✓ |  |  |  | ✓ |
| Q. Quality of Information | Quality of information is too broad. This question should be split into clarity and appropriateness. |  | ✓ |  | ✓ |  |
| Q. Quality of Information | Poor. Some of the information is provided in a clear and appropriate way. 3. Fair. The information is provided in a way that is sometimes clear and appropriate, but could be improved. 4. Good. The information is provided in a way that is usually clear and appropriate for the target audience, but still not ideal. | ✓ |  |  | ✓ |  |
| Q. Complete & concise | These are two different dimensions | ✓ |  |  |  | ✓ |
| Q. Clarity | Fair. There are some explanations as to the program’s purpose, but these are often insufficient. | ✓ |  |  | ✓ |  |
| *Other comments received at the end of this section* | I would make appropriateness from question 20 a stand-alone question and include cultural appropriateness as one of the explanation. Others could include appropriateness in terms of target age group, literacy of the target group etc. | ✓ |  |  | ✓ |  |
|  | Cultural appropriateness will help improve usability or redesign of the framework |  |  |  |  |  |
| **Section 5: Therapeutic Persuasiveness** |  |  |  |  |  |  |
| Q. Call to action | "Motivate" would be a better health promotion language to use | ✓ |  |  | ✓ |  |
| Q. Real data driven | Not sure this is the right name | ✓ |  |  |  | ✓ |
| Q. Ongoing feedback | Could this be merged with rewards ? |  | ✓ |  |  | ✓ |
| Q. Expectations & relevance | Should this be split into 2 questions. |  | ✓ |  |  | ✓ |
| Q. Expectations & relevance | Not sure what is meant by "advocate for relevance". | ✓ |  |  |  | ✓ |
| Q. Expectations & relevance | The questions in this should not contradict the questions on motivating the interest of the user to use it. |  |  | ✓ |  | ✓ |
| Q. Expectations & relevance | Good. The EHP advocates for its relevance, and explains the framework and general expectations appropriately (but it could be improved). | ✓ |  |  | ✓ |  |
| *Other comments received at the end of this section* | The term system and program are used interchangeably? | ✓ |  |  | ✓ |  |
| **Section 6: Therapeutic Alliance** |  |  |  |  |  |  |
| Q. Basic acceptance | The adjective "basic" is unnecessary | ✓ |  |  |  | ✓ |
| *Other comments received at the end of this section* | Very important in motivating the user and feel of inclusiveness |  |  |  |  |  |
| **Section 7: General Subjective Evaluation** |  |  |  |  |  |  |
| Q. Appropriate features | I would suggest 'potential' therapeutic goals | ✓ |  |  | ✓ |  |
| Q. Right mix | This looks like a repetition of a previous question. I would just leave it at motivation and exclude right mix as this is very vague |  | ✓ |  |  | ✓ |
| Q. Right mix | This question is needed but needs focus? Is the initial question: to what degree will the target audience be able and motivated to utilize the program to reach the therapeutic aim? (standard Likert options). |  | ✓ |  |  | ✓ |
| Q. Right mix | Again 'potential; therapeutic aim | ✓ |  |  | ✓ |  |
| Q. I like the program | Do not really like it. | ✓ |  |  | ✓ |  |
| Q. I like the program | Q39. The responses should have reasons attached , they will help improve the framework such: 1. Do not like it all because.................... and for all the 5 questions. |  | ✓ |  |  | ✓ |
| *Other comments received at the end of this section* | You can also reword on "I like the program". | ✓ |  |  | ✓ |  |
|  | I would consider the impact of hearing loss especially age related hearing loss if audio files were used in the app |  |  | ✓ |  | ✓ |
| **Section 8: Checklists** |  |  |  |  |  |  |
| Q. Credibility | Just wondering why some of these criteria are weighted as 1 and others as 2. |  |  |  |  |  |
| Q. Credibility | I  think target group involvement in development, maintenance etc is important to be part of this in line with public/patient involvement in research e.g. https://www.hrb.ie/funding/funding-schemes/public-and-patient-involvement-in-research/ |  |  |  |  |  |
| Q. Evidenced based content | Just wondering why some of these criteria are weighted as 1 and others as 2. |  |  |  |  |  |
| Q. Privacy explanation | Just wondering why some of these criteria are weighted as 1 and others as 2. |  |  |  |  |  |
| Q. Privacy explanation | Statement that GDPR regulations met and is regulated by same considering variance across various countries |  |  |  |  |  |
| Q. Security | Just wondering why some of these criteria are weighted as 1 and others as 2. |  |  |  |  |  |
| **Other comments for the entirety of round I** |  |  |  |  |  |  |
|  | Very complex and boring tool. It should be made more user friendly. |  |  |  |  |  |
|  | There are some words which seem to be strong as suggested in the questionnaire. If those can be revised but maintain the meaning. |  |  |  |  |  |
|  | One potential end-user is a carer. Overall, the language used throughout is inconsistent. More thought is needed concerning terminology. Some indication of how much effort was spent assessing the App would be useful. |  |  |  |  |  |
|  | The categories are rather broad though the technology has the capacity to comprehend and address the needs of an individual user. |  |  |  |  |  |
|  | My mind concerns relate to target audiences with respect to sufficient appropriateness for literacy levels, visual and hearing impairment, cognitive and emotive levels. |  |  |  |  |  |
