# Supplementary material for: Modification and Validation of an mHealth App Quality Assessment Methodology for International Use: Cross-sectional and eDelphi Studies
Source: JMIR Form Res. 2022 Aug 19;6(8):e36912. doi: 10.2196/36912 (PMC9497647; doi:10.2196/36912)
Supplement: Multimedia Appendix 2 [file formative_v6i8e36912_app2.docx]

**MODIFIED ENLIGHT SUMMARY REPORT OF [mHealth app Name]**

****** To be used in conjunction with the Enlight Checklist section******

**Classification**

Date of evaluation: ________________

# Version examined: ________________

Available on:  iPhone  Android

Evaluated on:  iPhone  Android

Program cost:  Free /________

Languages: ________________

**Clinical Classification**

**Target Audience Age Range** (in years): _____________ **Intended Users** (Mark all that apply)

 Clinician Consumer Parent/Children/Sibling/Concerned Significant Other  Researcher

**Clinical Condition** (Mark all that apply)

 Addiction-Related (e.g., alcohol, smoking, gambling, other substance abuse)
 Chronic Disease (e.g., asthma, cardiovascular disease, diabetes, pain, traumatic brain injury)

 Health related Behaviours (e.g., diet/weight loss, nutrition, physical activity, sleep/insomnia)
 Mental Health (e.g., anxiety, depression, disruptive behaviours, eating disorders, psychosis)
 Well-Being (e.g., emotional support, mindfulness, positive psychology, relationships)

**Program Aim** (Mark all that apply)

 Assessment
 Monitoring
 Awareness/Education
 Adherence to Treatment £ Health Care Management £ Prevention
 Treatment

**Special needs of the target audience taken into account during examination process:**(e.g., depression: lack of energy/motivation; elderly: simple layout/interface)

__________________________________________________________________________________

__________________________________________________________________________________

**Program description:** ____________________________________________________________________________________________________________________________________________________________________

**Concepts Description**

| Concepts | Description |
| --- | --- |
| **Quality Assessment Section** |  |
| Usability | Assesses the ease of learning how to use the app and the ease of utilizing it properly. |
| Visual Design | Assesses the look and feel of the app and its visual quality |
| User Engagement | Assesses the extent to which the app’s design attracts users to utilize it. |
| Content | Assesses the content provided or learned while using the app. |
| Therapeutic Persuasiveness | Assesses the extent to which the app is designed to encourage users to make positive behaviour changes OR to maintain positive aspects of their life. |
| Therapeutic Alliance | Assesses the ability of the app to create an alliance with the user to effect a beneficial change. |
| General Subjective Evaluation of app’s Potential | Examines the app’s general potential to benefit its target audience based on the rater’s subjective evaluation. |

**Usability**

***Assesses the ease of learning how to use the app and the ease of utilizing it properly.*
*Note: Slow speed of operation should be reflected in all items.***

**A. Navigation.** Is it easy/natural/intuitive to navigate through the app?
Note: Pay attention to how easy it is to (a) move from one location to another (if needed), and (b) move backwards.
**1. Very poor.** It is very difficult to move from one place to another. Many features are, therefore, not easily accessible when needed.
**2. Poor.** It is difficult to move from one place to another, making some features somewhat hard to reach when needed.
**3. Fair.** Navigation is okay, but not smooth.
**4. Good.** It is simple/natural to navigate through the app flow (but not ideal).
**5. Very good.** It is very clear how to navigate through the app and to access every desired / relevant location when needed.

**B. Learnability.** How easy is it to learn how to use the app at first? Is it self-explanatory/intuitive? Note: Consider complexity. Some apps are very complex and so might only score a maximum of 4.
**1. Very poor.** It takes too much time to learn how to use the app.
**2. Poor.** It takes a considerable amount of time to learn how to use the app. Only highly motivated users will take the time to learn it OR supplementary support is needed.
**3. Fair.** Users can learn how to use the app without additional support. Only a few of the important features require a considerable amount of time to learn.
**4. Good.** Learning to use the app is easy (but not ideal). Appropriate explanations appear if needed. None of the important features require a considerable amount of time to learn.
**5. Very good.** Learning to use the app is very easy, natural, and intuitive.

**C. Ease of Use.** How easy is it to use the app? Does the user need to exert minimal effort to activate the desired features?
**1. Very poor.** The user has to exert a lot of effort that would have been unnecessary had the app been better designed. **2. Poor.** Utilizing key parts of the app demands effort from the end user.
**3. Fair.** Utilizing some parts of the app demands effort from the end user.
**4. Good.** Utilization could have been made more effortless by better designing one (not major) feature.
**5. Very good.** The design best minimizes the effort required from the user.

**D. Timeliness.** How accurately/fast do the app features (functions) and components (buttons/menus) work?
**1. Very Poor.** App is broken; no/insufficient/inaccurate response (e.g. crashes/bugs/broken features, etc.)
**2. Poor.** Some functions work, but lagging or contains major technical problems
**3. Fair.** App works overall. Some technical problems need fixing, or is slow at times
**4. Good.** Mostly functional with minor/negligible problems
**5. Very Good.** Perfect/timely response; no technical bugs found, or contains a ‘loading time left’ indicator (if relevant)

**E. Errors:** Were errors (such as stalling, crashing, failing to save information or app connectivity issues) encountered whilst using the app?
**1. Very Poor.** A couple of major errors were encountered. As a result it is highly likely that the app would be deleted.
**2. Poor.** A couple of minor errors (e.g. 3) were encountered. Only highly motivated users would likely continue to use the app.
**3. Fair.** Some minor errors (e.g., <3) were encountered.
**4. Good.** Very few minor errors (e.g. 1) were encountered. This error does not have a significant impact on the use of the app.
**5. Very Good.** No errors were encountered.

**F. Understandability.** To what degree would the average person be able to understand the information contained within this app?
**1. Very poor.** The information within this app is written in an excessively difficult manner - Medical Jargon is evident.
**2. Poor.** Some medical jargon is present.
**3. Fair.** The information within the app is relatively easy to comprehend, however a certain degree of doubt is present.
**4. Good.** Most of the Information within this app is readily understandable to the average user.
**5. Very Good.** Information within this app is explained in everyday terms - All information within this app would be understood by the average user.

**G. Access.** Are you guaranteed access to the app at any time?
**1. Very Poor.** The app does not facilitate an offline mode.
**2. Poor.** The app does facilitate an offline mode, albeit with extremely limited functionalities.
**3. Fair.** The app does facilitate an offline mode, albeit with limited functionalities.
**4. Good.** Comprehensive features are available in offline mode. Manual syncing only.
**5. Very Good.** Comprehensive features are available in offline mode. Syncing occurs automatically when back online.

**Visual Design**

***Assesses the look and feel of the App, and its visual quality.***

**A. Aesthetics.** Is the interface design of the app attractive and appealing to its target audience? Does the app have a harmonious look and feel (including colours and fonts)?
**1. Not attractive at all.** The choice of colours/fonts/background is very poor.
**2. Not attractive.** The choice of colours/fonts/background does not make sense; however, some things are still adequately
designed.
**3. Fair.** The interface design makes some sense, but it is not attractive.
**4. Attractive.** Most parts of the interface design are attractive but could be improved.
**5. Very attractive.** The interface design is well thought-out, and the app has a harmonious look and feel.

**B. Layout.** Does the app appear well-organized? Relate to: structure (e.g., pattern, consistency); how well it displays (only necessary) elements on screen; and whether important information is clear and stands out.
**1. Very poor.** The basic layout is completely wrong and disorganized. Frames don’t fit the screen, and important parts are not featured.
**2. Poor.** The basic layout is poor and disorganized, but some aspects are adequate.
**3. Fair.** For the most part, relevant elements appear on the screen and more important aspects are featured. However, there are
still some issues with the apps structure and organization.
**4. Good.** In addition to ensuring the relevance and salience of key aspects, the app is also structured and organized.
**5. Very good.** The app is very well structured and organized. Elements are displayed appropriately.

**C. Size.** Are the sizes of fonts/buttons/menus appropriate (for the target audience)? Can the size be changed if needed?
**1. Very poor.** All fonts/features are inappropriately sized. Things do not fit the screen (navigation / scrolling is needed) for no apparent reason.
**2. Poor.** The main fonts/features are inappropriately sized.
**3. Fair.** There is at least one key place where the size is wrong.
**4. Good.** Most of the fonts/buttons/menus are appropriately sized, but there is at least one place where the size is wrong.
**5. Very good.** All fonts/buttons/menus are appropriately sized and well thought-out.

**User Engagement**

***Assesses the extent to which the App’s design attracts users to utilize it.***

**A. Content Presentation.** Is the content presented in an engaging/interesting way (e.g., contains the right mix of video/audio/text/graphics)?
**1. Very poor.** The content is poorly presented throughout. For example, there is often text where narration would be more appropriate.
**2. Poor.** For the most part, the content is presented poorly, but some areas of presentation are adequate.
**3. Fair.** Some major areas are presented appropriately (e.g., via text or audio), but better ways to present the content are still needed.
**4. Good.** Content is delivered through an appropriate combination of features, but could be improved.
**5. Very good.** The content is presented in an engaging/interesting way.

B. I**nteractive.** Does the app include high-quality interactive features (which enable user input and reaction)?
**1. Very poor.** There are no interactive features.
**2. Poor.** There are few interactive features, or the interactive features are of poor quality.
**3. Fair.** There are interactive features, but they are of mediocre quality.
**4. Good.** The app presents a good interactive experience (but there is room for improvement).
**5. Very good.** The app presents a high-quality interactive experience.
**N/A –** The app is not (highly) interactive, and so this is not an appropriate way to examine such a program, for example, a trigger-based intervention.

**C. Not Irritating.** Does the app avoid irritation in the user’s experience (e.g., by controlling notifications/alerts/sounds or avoiding irritating colours/fonts/sounds/expressions)? Note: Consider pop-up advertisements.
**1. Very poor.** It is annoying and irritating to utilize the app.
**2. Poor.** Some of the app’s key features are irritating.
**3. Fair.** There are some irritating features.
**4. Good.** For the most part, the app is not irritating, and users are able to modify any irritating aspects.
**5. Very good.** The app is not at all irritating, and, if relevant, users are given the opportunity to control potentially irritating aspects at the outset in order to avoid an irritating experience.
**N/A –** The app is not irritating, and this is not an appropriate way to examine such a program. For example, an app is very lean (e.g., absent of reminders that it should have) and therefore does not have the potential to be irritating (i.e., giving it a high score would inappropriately skew the results).

**C. Targeted/Tailored/Personalized.** Are the features tailored to the usage context of the target group? If necessary, does the app enable the customization/personalization (e.g., personalized goals/action items, online diary that maintains personal notes, choice of which content to receive)?
**1. Very poor.** The app does not have any targeted/tailored/personalized features.
**2. Poor.** The app includes a few targeted/tailored/personalized features.
**3. Fair.** The app incorporates a fair amount of targeting/tailoring/personalization.
**4. Good.** The app mostly provides a tailored/personalized experience based on users’ needs.
**5. Very good.** The app is very well designed in terms of offering the user a targeted/tailored/personalized experience.

**D. Captivating.** Does utilizing the app engage the user’s curiosity and interest (i.e., attract users to use it as needed)?
**1. Very poor.** The app is extremely boring and not desirable to use.
**2. Poor.** For the most part, the app features are boring, but there are some positives.
**3. Fair.** The app is neither boring, nor captivating.
**4. Good.** The app is interesting to use.
**5. Very good.** The app is highly attractive and engages the user’s curiosity, excitement, and interest.

**Content**
***Assesses the content provided or learned while using the app.
Note: As features (e.g., games) are a way of delivering information, the content conveyed within them should be examined.***

**A. Evidence-Based Content.** Is the information provided accurate? Are there evidence-based techniques relevant for achieving the desired clinical aim of the app?
**1. Very poor.** The features/content do not reflect any evidence-based principles in this field.
**2. Poor.** The presentation of evidence-based techniques is sparse OR the app content is not very accurate.
**3. Fair.** There is some presentation of evidence-based techniques, and the content is mostly accurate.
**4. Good.** The app content is accurate and reflects evidence-based techniques (but is still not ideal).
**5. Very good.** The app content is accurate and based on sound evidence-based principles relevant to the clinical aim.

**B. Quality of Information Provision.** Is the information provided clearly for the target audience? Note: Users’ age and cognitive and emotional abilities should be taken into account.
**1. Very poor.** None of the information is provided clearly for the target audience.
**2. Poor.** Some of the information is provided clearly.
**3. Fair.** The information is provided in a clear way but could be better.
**4. Good.** The information is provided clearly for the target audience, but still not ideal.
**5. Very good.** The information is provided in the most-clear way possible for the target audience.

**C.** **Complete and Concise.** Is there sufficient information throughout the app without any omissions, over-explanations, or irrelevant data?
**1. Very poor.** There is too much content that does not allow the user to grasp the relevant information, OR there is almost no content.
**2. Poor.** There is a great deal of content that interferes with the relevant information, OR the content is sparse.
**3. Fair.** There is some superfluous information, OR there are some omissions.
**4. Good.** The information is complete, but not concise enough, OR the information is concise, but not entirely complete.
**5. Very good.** The content is as complete and concise as it can be.

**D. Clarity about the app's purpose.** Is there sufficient and accurate information about the target audience, the clinical aim (e.g., potential outcomes), and appropriate ways to utilize the app (e.g., adjunct, standalone)? Notes: Includes who should not use it; could be described in distribution channels such as app stores.
**1. Very poor.** There is no information at all about the app's purpose. / Information is either inappropriate or inaccurate.
**2. Poor.** There is little information or poor accuracy.
**3. Fair.** There are some explanations as to the app's purpose, however these are often insufficient.
**4. Good.** The app explains who should use the program, what its purpose is, and how it should be utilized, but some information is still lacking.
**5. Very good.** The app provides a thorough explanation of who should use the program, what its purpose is, and how it should be utilized.

**E. Cultural appropriateness:** Does the app convey a message in a manner appropriate for its target audience? Note: Users age, Cognitive and Emotional Abilities should be taken into account.
**1. Very Poor.** None of the information is appropriate for the apps target audience. A cultural match is not evident.
**2. Poor.** Some of the information is appropriate for its target audience. Some images / examples are not be suitable.
**3. Fair.** The information within the app could be more appropriate for its target audience. A fair degree of cultural match is evident.
**4. Good.** The information within the app is appropriate for its target audience, but not still ideal. Some images / examples could be improved.
**5. Very Good.** The information within the app is appropriate for its target audience.

**Therapeutic Persuasiveness**
***Assesses the extent to which the app is designed to encourage users to make positive behaviour changes OR to maintain positive aspects of their life.***
***Note: Factors of social support (e.g., influence, facilitation, cooperation, recognition) should be taken into account while rating.***

**A. Call to Action.** Does the app easily set up measurable and relevant therapeutic activities and inspire/encourage/motivate users to complete them?
Notes: Includes sending out prompts if appropriate; does the user have to take part in the goal setting for the desired action(s) to be relevant/agreeable in this app? If so, rate accordingly.
**1. Very Poor.** Action items are vague, implied, hidden, or non-existent.
**2. Poor.** Some action items exist, but the app doesn't motivate users at all.
**3. Fair.** There are some relevant/targeted action items, and there is some degree of inspiration/encouragement/motivation.
**4. Good.** For the most part, there are relevant/targeted action items and the app stimulates/inspires/motivates users to meet their goals.
**5. Very good.** The desired therapeutic activities are well targeted, and the app clearly stimulates/inspires/motivates users to complete the activities.

**B. Therapeutic Rationale and Pathway.** Is the therapeutic pathway clear? Is it clear how working through each action item provided by the app should lead to the desired therapeutic outcome(s)? Note: This should also be considered from the user’s perspective.
**1. Very poor.** Users are asked to engage in activities without the therapeutic pathway being defined. The relationship between the activities and the desired outcome does not make sense.
**2. Poor.** While the relationship between the activities and therapeutic progress is understood, it is not clear how the app design and the way the action items are provided should lead to the desired therapeutic outcome.
**3. Fair.** It is somewhat clear how the app design and the way the action items are provided should lead to the desired therapeutic outcome.
**4. Good**. It is clear how the App design and the way the action items are provided should lead to the desired therapeutic outcome (but still not ideal).
**5. Very good.** It is very clear how the app design and the way the action items are provided should lead to the desired therapeutic outcome.

**C. Rewards.** Does the app recognize desirable achievements and provide appropriate recognition?
Note: This includes documentation of ”therapeutic investments,” i.e., beneficial work done by the user that is documented in the app in a way that makes users want to stay committed to this pathway (e.g., acquiring points/badges for beneficial activities and showing them on a community board).
**1. Very poor.** The app does not reward users at all.
**2. Poor.** The app uses rewards sparsely/inappropriately.
**3. Fair.** The frequency/appropriateness of rewards is only average.
**4. Good.** The app pays attention to desirable achievements. There are rewards most of the time, but they are not ideal (e.g., the same rewards are used all the time, too many rewards, or rewards not creative/accurate enough).
**5. Very good.** The app does a very good job acknowledging when users reach desirable achievements and rewarding them appropriately/creatively/accurately.

**D. Real Data-Driven/Adaptive Content.** Is the app content influenced by the end user’s state and/or achievements? Examples: Content becomes available when the user is ready (i.e., has made appropriate progress); app content changes based on the user’s real behaviour/success/failures.
Note: The user’s state does not have to rely on self-assessment; other methods could include passive sensing and clinicians’ input.
**1. Very poor.** The user’s progress is not monitored, and content is available regardless of the user’s state.
**2. Poor.** The user’s progress is not well monitored, and content mostly disregards the user’s state.
**3. Fair.** The user’s progress is monitored but not in a way that has a strong impact on app content, OR the app is adaptive, but not based on an accurate evaluation of the user’s state.
**4. Good.** The app appropriately monitors the user’s state and relies somewhat on the user’s progress to determine content.
**5. Very good.** The app adapts well to the user’s state/progress by changing its available content accordingly.

**E.** **Ongoing Feedback.** Does the app provide appropriate ongoing feedback on the user’s state?
**1. Very poor.** The app does not provide any feedback.
**2. Poor.** The app provides minimal feedback, for example, only after enrolment and taking baseline measurements.
**3. Fair.** Feedback is embedded within the app (e.g., graphs of outcome measures, calorie intake), but not in a way that provides users with a good understanding of their state.
**4. Good.** Feedback is embedded within the app, mainly in a way that provides users with an understanding of their state (e.g., via clear verbal explanation).
**5. Very good.** Feedback is embedded within the app with salient, accurate, and appropriate regard to the user’s current state.

**F. Expectations and Relevance.** Does the app convincingly advocate for intervention’s relevance, and explain the intervention framework and the general expectations of the user?
Note: Advocating entails relating to one’s own state, difficulties in making/sustaining a change, motivation and consequences for using it.
**1. Very poor.** There is no explanation of the app’s relevance and its expectations of the user.
**2. Poor.** The app offers only limited explanation of its relevance and expectations of the user.
**3. Fair.** The app offers an adequate explanation of its relevance and expectations of the user.
**4. Good.** The app advocates for its relevance, and explains the framework and general expectations appropriately (but it could be improved).
**5. Very good.** The app effectively advocates for its relevance, and explains the framework and general expectations.
**N/A –** The app does not explain its expectations/relevance, but this is not an appropriate way to examine such a program. For example, the targeting of an App makes it irrelevant to set up expectations.

**Therapeutic Alliance
*Assesses the ability of the app to create an alliance with the user in order to effect a beneficial change. Note: Factors of social support (e.g., influence, facilitation, cooperation, recognition) should be taken into account while rating.***

***A.* Acceptance and Support.** Does the app make an effort to show that it understands and empathizes with the user; genuinely cares for the user; and relates to the user in a positive fashion?
Note: The app is not a person so this should be done appropriately within the limits of the medium.
**1. Very poor.** There is no positive regard for OR effort to understand the user’s perspective.
**2. Poor.** There is only a minimal gesture to demonstrate understanding/caring for the user’s perspective.
**3. Fair.** In general, there is positive regard and care for the user (some degree of outreach is needed to receive 3).
**4. Good.** The app is designed to provide users with feelings of basic acceptance and support.
**5. Very good.** The app proactively shows users that they are accepted and supported as a salient aspect of the App.

**B**. **Positive Therapeutic Expectations.** Does the app encourage users to expect beneficial outcomes from utilizing the program and to rely upon it in the medical context?
Note: Consider how well the app instils confidence in users that they are in “good hands” (projecting trustworthiness and professionalism through tone, narrative, convincing presentation, reliable “look and feel”, and meeting people’s exact needs at the right time).
**1. Very poor.** The app does not instil confidence in users that they will benefit from the program. No professionalism/trustworthiness is conveyed.
**2. Poor.** The app instils minimal confidence in the user and conveys limited professionalism/trustworthiness.
**3. Fair.** The app instils some confidence in the user and conveys some professionalism/trustworthiness.
**4. Good.** The app instils a good degree of confidence in the user and conveys a good degree of professionalism/trustworthiness, but something is still missing.
**5. Very good.** The app effectively instils confidence in users that they will benefit from the program through professionalism and trustworthiness.

**C. Relatability.** Does the app offer a good representation of a human factor that is easily relatable within the therapeutic context/process? Examples include a professional who directs the user throughout the program; a peer who was in a similar situation and is now better (e.g., fitness); a vivid virtual character who leads the user; a community of people working together for change.
Notes: A community of people NOT “working” to positively support each other does not count; even text messages could create such projections through language, sender’s identity, and responsiveness.
**1. Very poor.** There is no relatable human factor.
**2. Poor.** Some representation of a human factor exists, but it is not really therapeutic or easily relatable.
**3. Fair.** There is a representation of a positive human factor, but no effort is made to communicate with the user on a personal level. The human factor seems somewhat distant from the user.
**4. Good.** There is a representation of a human factor that users can relate to throughout the therapeutic process. However, users might not be able to relate to this factor in an ideal way.
**5. Very good.** The representation of a human factor is salient throughout the therapeutic process; for example, users are potentially able to become really familiar with this human factor (e.g., professional character) or feel they are part of a community.

**General Subjective Evaluation of Program’s Potential**
***Examines the app’s general potential to benefit its target audience based on rater’s subjective evaluation.***

**A. Appropriate Features to Meet the Clinical Aim.** Are the apps features sufficient enough to meet its potential therapeutic goals?
1. Not at all.
2. Mostly not.
3. To some extent.
4. Appropriate.
5. Very appropriate.

**B. Right Mix of Ability and Motivation.** Is the target audience able and motivated to utilize the app as much as needed to reach the potential therapeutic aim?
Note: A change is created when people are able and motivated enough to make the change. If the change is easy, motivation doesn’t have to be as high, and vice versa.
1. Not the right mix at all.
 2. Mostly not the right mix.
3. To some extent.
4. Good mix.
5. Excellent mix.

**C. I Like the app?**1. Do not like it at all.
2. Do not really like it.
3. Like it to some extent
4. Like the app.
5. Like the app very much.
