# Supplementary material for: Modification and Validation of an mHealth App Quality Assessment Methodology for International Use: Cross-sectional and eDelphi Studies
Source: JMIR Form Res. 2022 Aug 19;6(8):e36912. doi: 10.2196/36912 (PMC9497647; doi:10.2196/36912)
Supplement: Multimedia Appendix 3 [file formative_v6i8e36912_app3.docx]

| **Delphi process: Round II** |  |  |  |  |  |
| --- | --- | --- | --- | --- | --- |
|  |  | **Classification** | | **Decision** | |
|  | **Amendments** | **Minor** | **Other** | **Accept** | **Reject** |
| **Section 1: Useability** |  |  |  |  |  |
| Q. Navigation | Enhances clarity |  |  |  |  |
| Q. Ease of use | Would substituting 'minimal' for 'only the least possible' assist? | ✓ |  | ✓ |  |
| Q. Errors | Great wording |  |  |  |  |
| Q. Accessibility | Can it read "Are you guaranteed to access the App at any time? | ✓ |  | ✓ |  |
| Q. Accessibility | To maintain parity of wording with the subsequent option substitute 'the app does not work offline' for 'the app does not facilitate an offline mode' | ✓ |  | ✓ |  |
| *Other comments received at the end of this section* | Can we change "App" to small letters please. "app" | ✓ |  | ✓ |  |
| **Section 2: Visual Design** |  |  |  |  |  |
| Q. Layout | Language consistency between 4 and 5? (Structured, organised), (organised, prioritised) | ✓ |  | ✓ |  |
| Q. Size | The wording of options 3 and 4 seem identical | ✓ |  |  | ✓ |
| **Section 3: User Engagement** |  |  |  |  |  |
| Q. Not irritating | This question feels a bit leading, and I am not sure what the purpose of it is compared to the other questions? |  | ✓ |  | ✓ |
| Q. Captivating | Good question - is 'captivating' an easy-to-understand word? | ✓ |  |  | ✓ |
| **Section 4: Content** |  |  |  |  |  |
| Q. Quality of information | Would 'clearly' read more easily than 'in a clear way'? | ✓ |  | ✓ |  |
| Q. Culture appropriateness | Standardize on hyphen or full stop. | ✓ |  | ✓ |  |
| *Other comments received at the end of this section* | These questions are to the point |  |  |  |  |
| **Section 5: Therapeutic Persuasiveness** |  |  |  |  |  |
| Q. Call to action | Not sure about "saliently". | ✓ |  | ✓ |  |
| Q. Load reduction of activities | I am not sure if the question name matches the content options? Load reduction in what context? | ✓ |  |  | ✓ |
| Q. Rewards | I feel like the reward is not coming out clearly. |  | ✓ |  | ✓ |
| Q. Rewards | Change "fair" in option 3 explanation, possibly to average or equivalent. If rewards are "fair", well that is fair enough, right? | ✓ |  | ✓ |  |
| Q. Rewards | Note that you could include N/a. | ✓ |  |  | ✓ |
| Q. Rewards | ‘Rewards' is difficult to specify how would you show that a given feature is valued and thereby motivating to all the users? |  | ✓ |  |  |
| Q. Real data driven | Change "Real user" to end-user? or user? | ✓ |  | ✓ |  |
| *Other comments received at the end of this section* | There is some potential overlap and/or ambiguity but could be lived with I guess. |  | ✓ |  |  |
|  | I feel as though there is a tension here between who the end-user actually is. My understanding is that medical professionals will complete this survey and patients will use the corresponding App. Some questions (comments/responses) suggest that it is actually the medical professional who will be the end-user. |  | ✓ |  |  |
|  | All of these relate to key aspects of technology supporting successful healthcare interventions and increasing the potential for improved healthcare outcomes |  | ✓ |  |  |
| **Section 6: Therapeutic Alliance** |  |  |  |  |  |
| Q. Basic acceptance | Why is 'neutral' given with option 1 'very poor' | ✓ |  | ✓ |  |
| Q. Positive therapeutic expectations | Why is 'neutral' given with option 1 'very poor' | ✓ |  | ✓ |  |
| Q. Relatability | Would substituting 'character' for 'factor' (and omitting 'character' in line 2) assist the reader? | ✓ |  |  | ✓ |
| **Section 7: General Subjective Evaluation** |  |  |  |  |  |
| Q. Right mix | "Right mix" to "Appropriate mix"? | ✓ |  |  | ✓ |
| *Other comments received at the end of this section* | Age related hearing loss I would suggest is important for audio files and should be highlighted as an important aspect of usefulness if audio files are used in an app. While it may be outside the scope of questionnaire this maybe because no one has thought about it. Maybe should be an adjunct to questionnaire if significant audio file used in apps. "Age-related hearing loss and speech perception disorder: the broken interface between healthcare professionals and older adults" Editorial from European Geriatric Medicine August 2020 https://link.springer.com/article/10.1007%2Fs41999-020-00379-y |  | ✓ |  |  |
| **Other comments for the entirety of round II** | |  |  |  |  |
|  | I think it is great! |  |  |  |  |
|  | Some of the questions just need to be concise. |  |  |  |  |
|  | See my comments on age related hearing loss. |  |  |  |  |
|  | The changes were thoughtful and justified. |  |  |  |  |
